# Antibiotic use among young, hospitalized children in Jordan, 2010–2023

**DOI:** 10.1128/spectrum.02691-24

**Published:** 2025-02-14

**Authors:** Haya Hayek, Justin Z. Amarin, Olla Hamdan, Yasmeen Z. Qwaider, Tala Khraise, Ritu Banerjee, Andrew J. Spieker, James D. Chappell, Najwa Khuri-Bulos, Sophie E. Katz, Leigh M. Howard, Natasha B. Halasa, for the Jordan Viral Surveillance Studies Group

**Affiliations:** 1 Department of Pediatrics, Vanderbilt University Medical Center, Nashville, Tennessee, USA; 2 Epidemiology Doctoral Program, School of Medicine, Vanderbilt University, Nashville, Tennessee, USA; 3 Department of Biostatistics, Vanderbilt University Medical Center, Nashville, Tennessee, USA; 4 Department of Pediatrics, School of Medicine, The University of Jordan, Amman, Jordan; bioMerieux Inc, Denver, Colorado, USA

**Keywords:** antibacterial agents, antimicrobial stewardship, virus diseases

## Abstract

Inappropriate antibiotic use drives antimicrobial resistance, a global health threat causing increased morbidity and mortality. Understanding antibiotic practices in low-resource settings is essential to identify intervention targets. This study investigates antibiotic prescription practices in Amman, Jordan. We conducted three prospective viral surveillance studies at the largest public hospital in Amman, Jordan (2010–2013, 2020, and 2023) and included children <2 years old hospitalized with fever or respiratory symptoms. The data collected included antibiotic use and the results of blood, urine, and cerebrospinal fluid (CSF) cultures performed. We classified antibiotics according to the 2023 WHO AWaRe system. Nasal or throat samples were tested in a research laboratory for respiratory viruses using RT-PCR. The median age of the 4,724 children included was 3.5 months (IQR, 1.6–8.4). A clinical blood, urine, or CSF sample was collected from 2,565/4,712 children (54.4%), 356 (13.9%) of whom tested positive. During hospitalization, 4,375 children (92.6%) received at least one antibiotic, and 4,245 (97.0%) received at least one antibiotic from the *Watch* group. One or more respiratory viruses were detected in 3,911 children (82.8%). Providers ordered cultures most often for children 0–2 months old (*n* = 1,579 [73.5%]) and those with an admission diagnosis of rule-out sepsis (*n* = 1,164 [95.4%]). Antibiotic use and testing practices were consistent across study years, age groups, and admission diagnoses. In conclusion, widespread antibiotic use despite the preponderance of viral infections highlights a significant discrepancy in aligning treatment practices with disease etiology. Strengthening diagnostic and preventive capabilities in low-resource settings is crucial to combat antimicrobial resistance.

**IMPORTANCE:**

In this study of 4,724 children under 2 years old hospitalized in the largest public hospital in Jordan between 2010 and 2023, 92.6% received antibiotics despite 82.8% testing positive for respiratory viruses and only 13.9% of collected cultures suggesting bacterial infection. Despite the predominance of viral infections, the widespread use of antibiotics, particularly from the World Health Organization Watch group, highlights the need for improved antibiotic stewardship and diagnostic capabilities in Jordan.

## INTRODUCTION

Antimicrobial resistance (AMR) represents an escalating global health threat, leading to increased morbidity, mortality, and healthcare costs (1). Misuse and overuse of antibiotics, primarily due to inappropriate prescription practices, are the major drivers of AMR (2). In low-resource settings, distinguishing bacterial from viral infections is challenging due to limited access to diagnostic tests (3). Previous studies indicate a persistent trend of inappropriate antibiotic use in low-resource settings, including Amman, Jordan (4). Pediatric populations are especially vulnerable, as antibiotic overprescription can lead to adverse health effects (5, 6). In Jordan, no other viral surveillance efforts have systematically collected granular clinical data, including antibiotic use practices, over an extended period. Understanding the factors influencing antibiotic use is imperative for reducing AMR in the region (7). We analyzed antibiotic use practices in Amman, Jordan, using the data collected from pediatric viral surveillance studies.

## MATERIALS AND METHODS

Data from three prospective viral surveillance studies conducted at Al-Bashir Hospital, Jordan’s largest public hospital, located in Amman, in 2010–2013, 2020, and 2023 were analyzed. The methods for the studies conducted in 2010–2013 and 2020 were previously reported (8, 9). In 2023, we enrolled hospitalized children who met the following criteria: age at admission <5 years, onset of fever or at least one respiratory symptom within 14 days prior to admission, and screening completed within 72 hours of admission. We excluded three groups: newborns who were never discharged home after birth, children enrolled in our study within the 14 days prior to admission, and those with a known nonrespiratory cause for hospitalization. To construct a homogeneous cohort across the studies, we included in this study children <2 years old who had nasal or throat swabs tested for respiratory viruses through research testing using RT-PCR and whose antibiotic history was available. The respiratory viruses included influenza, parainfluenza viruses (PIV-1–4), human rhinovirus/enterovirus (HRV/EV), adenovirus (AdV), respiratory syncytial virus (RSV), human metapneumovirus (HMPV), common cold coronaviruses (ccCoV), and SARS-CoV-2 (2020 and 2023). These research tests were performed weeks to months after sample collection and were not documented in the clinical charts. Demographic and clinical data, antibiotic use, and urine, blood, and cerebrospinal fluid culture results were obtained via parental interviews and clinical chart reviews. The 2023 World Health Organization (WHO) AWaRe (*A*ccess, *Wa*tch, *Re*serve) antibiotic classification system was used (10). The Watch group comprises antibiotics used when first-line treatments (Access) are not effective or appropriate. All positive microbial culture results were independently adjudicated by three pediatric infectious disease specialists (SEK, LMH, and NBH). The adjudicators used clinical judgment informed by pertinent clinical information to classify each positive culture as either “Probable infection,” “Possible contamination or colonization,” or “Unable to adjudicate” (nonvalidated scheme). Discrepancies were resolved by consensus. Using R (version 4.3.2), we described categorical variables using absolute and relative frequencies and summarized continuous variables using the median and interquartile range (IQR). The Vanderbilt University Institutional Review Board and the Jordanian Ministry of Health’s Institutional Review Board reviewed and approved the study protocols. Parents or legal guardians provided written informed consent.

## RESULTS

### Description of the cohort

We enrolled a total of 4,942 children in 2010 (*n* = 340), 2011 (*n* = 1,031), 2012 (*n* = 1,332), 2013 (*n* = 465), 2020 (*n* = 532), and 2023 (*n* = 1,242). We included 4,724 children (95.6%) eligible for this study. We describe the demographic and clinical characteristics of the study cohort in Table 1. Briefly, the median age was 3.5 months (IQR, 1.6–8.4), and males comprised 59.3% (*n* = 2,803) Most children were admitted to the general ward only (*n* = 4,323/4,713 [91.7%]), while 390/4,713 (8.3%) were admitted to the pediatric intensive care unit at least once. During hospitalization, 1,365/4,709 children (29.0%) received supplementary oxygen, 130/4,712 (2.8%) were intubated, and 39/4,707 (0.8%) died. The median length of stay was 5 days (IQR, 3–7; *n* = 11 missing observations). Of 1,644 children 6 months or older, 8/1,638 children (0.5%) had been vaccinated against seasonal influenza at presentation (by parental reporting).

**TABLE 1:** Demographic and clinical characteristics of the study cohort

Characteristic	Overall, *N* = 4,724
Age at admission (months)—median (IQR)	3.5 (1.6–8.4)
Sex—*n* (%)	
Female	1,921 (40.7)
Male	2,803 (59.3)
At least one underlying medical condition—*n* (%)	718 (15.2)
Born prematurely—*n* (%)	703 (14.9)
Currently breastfed—*n* (%)	339/529 (64.1)
Cough—*n* (%)	3,623 (76.7)
Shortness of breath—*n* (%)	2,884/4,722 (61.1)
Wheezing—*n* (%)	2,724/4,723 (57.7)
Fever—*n* (%)	2,681/4,714 (56.9)
Congestion or runny nose—*n* (%)	1,307/4,723 (27.7)
Vomiting—*n* (%)	1,163/4,723 (24.6)
Diarrhea—*n* (%)	879/4,722 (18.6)

### Pathogen testing

**Fig 1:**
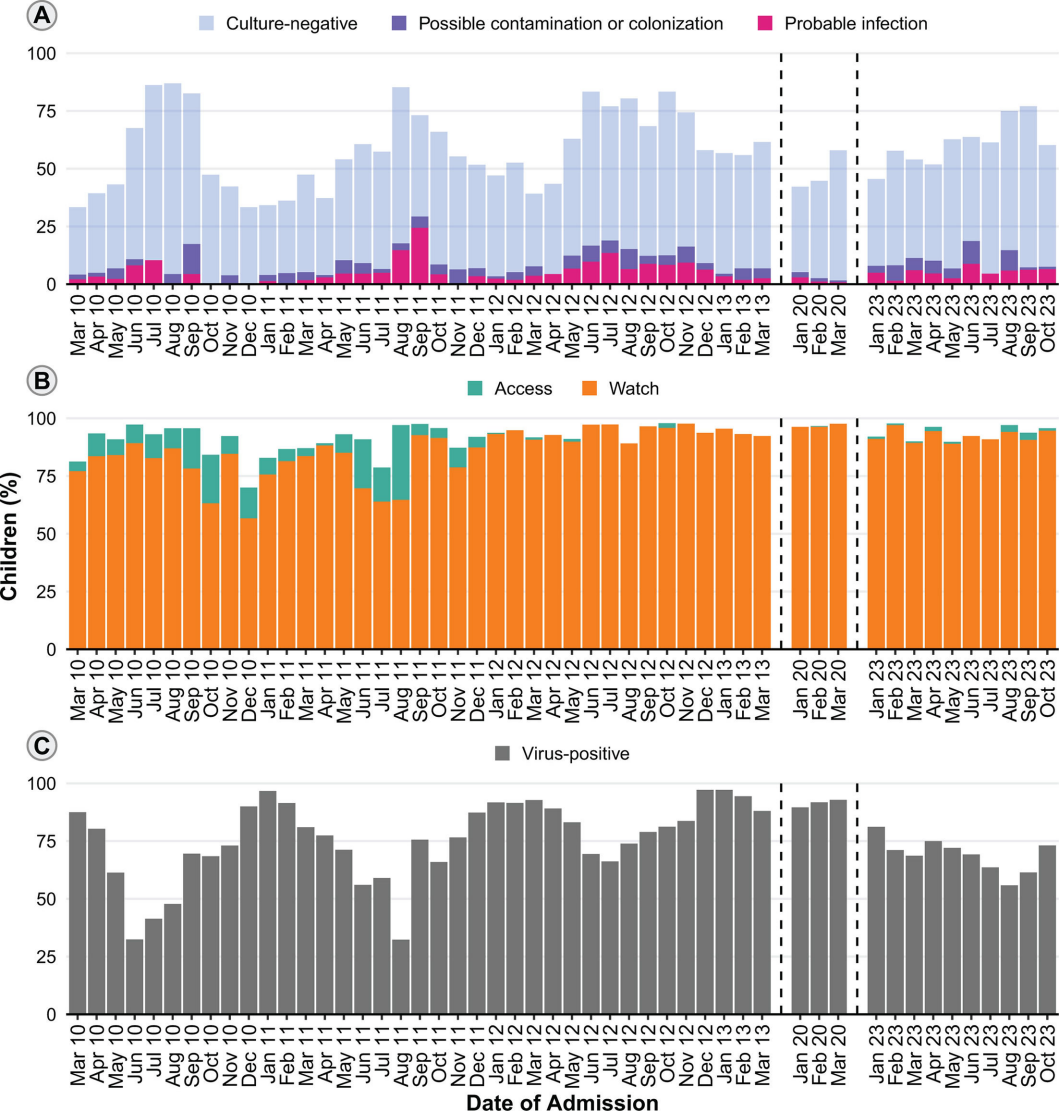
Culture results, antibiotic use, and viral testing results. (**A**) Bar plot of performed cultures (blood, urine, or cerebrospinal fluid) and positive results. (**B**) Bar plot showing antibiotic use distribution according to the World Health Organization AWaRe groups. (**C**) Bar plot indicating the proportion of children with at least one positive respiratory virus.

By research testing, 3,911 children (82.8%) were positive for at least one respiratory virus, of whom 1,378 (35.2%) were positive for more than one. The most common virus detected was RSV (*n* = 1,816 [38.4%]), followed by HRV/EV (*n* = 1,748 [37.0%]), AdV (*n* = 652 [13.8%]), HMPV (*n* = 402 [8.5%]), PIV (*n* = 341 [7.2%]), ccCoV (*n* = 276 [5.8%]), influenza (*n* = 190 [4.0%]), and SARS-CoV-2 (*n* = 115 [2.4%]). The information on whether a culture was collected was available for 4,712 children. A clinical sample of blood, urine, or CSF was collected from 2,565/4,712 (54.4%), 356 (13.9%) of whom tested positive for at least one bacterial pathogen (Table 2). Of these 356 positive cultures, 190/355 (53.5%) represented a probable bacterial infection by adjudication. Monthly culture and viral testing results are shown in Fig. 1A and C.

**TABLE 2:** Culture results of clinical samples of blood, urine, or cerebrospinal fluid (CSF)

Sample	*N* (%)
Any	2,565/4,712 (54.4)
Blood	2,207/4,712 (46.8)
Positive	166/2,207 (7.5)
Probable infection	50/166 (30.1)
Negative	2,041/2,207 (92.5)
Urine	1,675/4,712 (35.5)
Positive	202/1,675 (12.1)
Probable infection	145/202 (71.8)
Negative	1,473/1,675 (87.9)
CSF	1,121/4,712 (23.8)
Positive	13/1,121 (1.2)
Probable infection	7/13 (53.8)
Negative	1108/1,121 (98.8)

### Antibiotics

During hospitalization, 4,375 children (92.6%) were prescribed antibiotics. Of those, 4,245 (97.0%) were prescribed at least one antibiotic from the WHO Watch group. The three most common Access group antibiotics prescribed were ampicillin (*n* = 823 [18.8%]), gentamicin (*n* = 97 [2.2%]), and amikacin (*n* = 87 [2.0%]), whereas the three most common Watch group antibiotics prescribed were ceftriaxone (*n* = 1,968 [45.0%]), cefuroxime (*n* = 1,625 [37.1%]), and azithromycin (*n* = 749 [17.1%]). Figure 1B depicts monthly antibiotic use. Among 1,491 children prescribed antibiotics in 2020 or 2023, the median duration of in-hospital treatment for the antibiotic that was continuously administered the longest was 5 days (IQR, 3–6). Before hospitalization, 1,831/4,697 children (39.0%) received antibiotics for the treatment of the presenting illness. Among the 2,866 children who did not receive antibiotics before hospitalization, 2,667 (93.1%) were prescribed antibiotics during their hospital stay.

### Subgroups by age and admission diagnosis

We examined culture collection, culture results, antibiotic use, and viral testing results in the cohort, stratified by age group and admission diagnosis (Table 3). Cultures were most often clinically ordered for children 0–2 months old (*n* = 1,579 [73.5%]) and those with an admission diagnosis of rule-out sepsis (*n* = 1,164 [95.4%]). Other measures were generally consistent across age groups and admission diagnoses.

**TABLE 3:** Culture results, antibiotic use, and viral testing results, disaggregated by age group and admission diagnoses

Subgroup	*n* (%)	Any culture collected, *n* (%)	Any positive culture, *n* (%)	Any antibiotic use, *n* (%)	Any Watch group antibiotic use, *n* (%)	Any viral detection, *n* (%)
Age group (months)						
0–2	2,149 (45.5)	1,579 (73.5)	257 (12.0)	2,024 (94.2)	1,910 (88.9)	1,695 (78.9)
3–5	931 (19.7)	382 (41.0)	34 (3.7)	841 (90.3)	834 (89.6)	825 (88.6)
6–11	918 (19.4)	333 (36.3)	35 (3.8)	835 (91.0)	827 (90.1)	784 (85.4)
12–23	726 (15.4)	271 (37.3)	30 (4.1)	675 (93.0)	674 (92.8)	607 (83.6)
Admission diagnosis^*a*^						
Pneumonia	1,752 (37.1)	853 (48.7)	82 (4.7)	1,714 (97.8)	1,702 (97.1)	1,569 (89.6)
Rule-out sepsis	1,220 (25.8)	1,164 (95.4)	212 (17.4)	1,189 (97.5)	1,085 (88.9)	843 (69.1)
Bronchiolitis	870 (18.4)	214 (24.6)	22 (2.5)	712 (81.8)	703 (80.8)	809 (93.0)
Paroxysmal coughing or pertussis-like cough	275 (5.8)	106 (38.5)	13 (4.7)	261 (94.9)	260 (94.5)	242 (88.0)
Asthma exacerbation or reactive airway disease	162 (3.4)	26 (16.0)	2 (1.2)	121 (74.7)	120 (74.1)	147 (90.7)

^*a*^ Subgroups defined by admission diagnoses are not mutually exclusive.

## DISCUSSION

Our report identifies several challenges affecting antibiotic use practices in Jordan. First, we showed that antibiotic use was disproportionately high despite limited evidence of bacterial infections. Second, the high prevalence of respiratory viral detections, alongside widespread antibiotic use (particularly from the WHO Watch group), indicates a notable mismatch between the etiology of disease and treatment practices, which was persistent across study years, age subgroups, and admission diagnoses. Finally, in terms of prevention, we found that almost all the children eligible for influenza vaccination in our cohort were unvaccinated.

Globally, pediatric healthcare facilities grapple with the challenge of identifying cases that may benefit from antibiotics, particularly in low-resource settings (11). Unfortunately, the effectiveness of antimicrobial stewardship programs implemented in most public hospitals in Jordan (in 2018) appears to have been suboptimal (12). In addition, there is a critical need for improved point-of-care viral diagnostic testing and more accurate diagnostic tools for bacterial infections. As of May 2024, respiratory pathogen panel testing is available but underutilized in Jordan, and rapid diagnostics are rarely used at Al-Bashir Hospital. Enhanced diagnostic capabilities may enable more precise targeting of antibiotic therapy, thereby reducing unnecessary prescriptions (13). Additionally, preventive measures, such as the routine use of influenza vaccines, RSV monoclonal antibodies, and the pneumococcal conjugate vaccine (which is not part of the national immunization program), could indirectly reduce antibiotic use.

Al-Bashir Hospital follows the NICE guidelines for the assessment and initial management of febrile children <5 years old, which emphasize the targeted use of antibiotics only for specific high-risk presentations (14). However, our findings demonstrate significant divergence from these guidelines in actual practice, with most children—regardless of age group or admission diagnosis—receiving antibiotics despite predominant viral etiologies. This gap between guidelines and practice likely reflects multiple factors, including diagnostic uncertainty in resource-limited settings, cultural expectations around antibiotic use, and historical prescribing patterns. Future research should explore specific barriers to guideline adherence in this context.

This study has several limitations. First, biases in data collection across different study years may impact the generalizability of these findings. However, the use of standardized data collection forms and consistent eligibility criteria across the study periods enhances the comparability of our findings. Second, the surveillance studies were conducted in a single hospital. This limitation is mitigated by the study locale, Al-Bashir Hospital, which is the largest public hospital in Jordan and serves 50–60% of the target population (15). Third, a substantial proportion of children received antibiotics before hospitalization, possibly diminishing bacterial culture sensitivity. Furthermore, we did not adjudicate pneumonia cases in children who did not have positive cultures, which may have led to an underestimation of bacterial infections in our cohort. We, nonetheless, showed that most of these children had evidence of a viral etiology—though bacterial co-infection remains probable (16). Finally, gaps between the study periods could weaken our insights into the evolution of antibiotic use practices and AMR patterns. Even so, the data collected during the observed periods show remarkably consistent patterns, which indicates enduring widespread use of antibiotics.

### Conclusion

Our findings highlight the pressing issue of widespread antibiotic use in pediatric patients in Amman, Jordan, mirroring challenges faced in similar low-resource settings. The findings emphasize the urgent need for stringent antibiotic use guidelines and the reassessment of established antimicrobial stewardship programs. Additionally, a stronger focus on targeted preventive strategies is essential. These measures aggregately form a multifaceted approach to combat AMR in Jordan and comparable settings.
